# Navigating pregnancy with cardiovascular disease: pathophysiology, risk stratification, and maternal–fetal outcomes

**DOI:** 10.55730/1300-0144.5940

**Published:** 2024-11-10

**Authors:** Osman TÜRKMEN, Serra AKAR İNAN

**Affiliations:** 1 Department of Obstetrics and Gynecology, Faculty of Medicine, Ankara Yıldırım Beyazıt University, Ankara, Turkiye; 2 Department of Obstetrics and Gynecology, Faculty of Medicine, University of Health Sciences, Ankara, Turkiye

**Keywords:** Cardiovascular disease, pregnancy, congenital heart disease, cardiomyopathy, hypertension

## Abstract

Pregnancy induces significant cardiovascular changes, including increases in heart rate, blood volume, and cardiac output, which may exacerbate existing heart conditions or unmask previously undiagnosed heart diseases. This review outlines the types of cardiovascular diseases that affect pregnant women, organized according to the modified World Health Organization risk classification. It covers key areas, including how pregnancy impacts the heart, common maternal and fetal complications, and effective management strategies. The review emphasizes the importance of preconception counseling, risk assessment, and continuous monitoring to ensure the best maternal and fetal outcomes. It also highlights practical guidelines for healthcare providers and discusses the latest research findings to improve clinical care for pregnant women with heart disease.

## 1. Introduction

It is critical to study heart diseases during pregnancy because of the unique demands pregnancy places on the cardiovascular system. This review aims to provide a comprehensive overview of the maternal cardiovascular risk stratifications during pregnancy as well as various types of heart diseases that can affect pregnant women. Additionally, it discusses maternal and fetal outcomes associated with heart disease during pregnancy.

## 2. Epidemiology

Cardiovascular disease (excluding hypertensive disorders) complicates approximately 1%–4% of all pregnancies with rates as high as 26.5% of pregnancy-related deaths in the US. This establishes cardiovascular disease as one of the leading causes of maternal mortality in high-income countries [1,2]. The prevalence of cardiovascular diseases among hospitalized pregnant patients increased from 9.2% in 2010 to 14.8% in 2019 [3].

Hypertensive disorders were the most common (seen in about 10% of pregnancies) cardiovascular conditions [4,5].

Obesity, chronic metabolic conditions, and advanced maternal age are increasingly common among pregnant women, thus elevating their risk of developing cardiovascular complications [6]. Moreover, a recent study highlighted that in over a decade there was a notable increase in the incidence of severe cases of heart disease categorized as class III or IV by the modified World Health Organization (mWHO) cardiovascular risk categorization in pregnant women [7].

Demographic and regional differences significantly influence the prevalence of heart diseases during pregnancy. In low-income countries, rheumatic valvular disease is a leading cause of cardiac complications in pregnant women, whereas congenital heart disease (CHD) is more prevalent in high-income countries. For example, a study conducted in India found that rheumatic heart disease (RHD) accounted for 62.5% of cardiac cases among pregnant women, while CHD accounted for 12.5% [8]. In contrast, a European registry reported that CHD made up 57% of cardiac cases in pregnant women, with valvular heart disease accounting for 29% [9].

## 3. Mechanisms of cardiovascular adaptations

Pregnancy induces significant cardiovascular changes to meet the increased nutritional demands of both the mother and the fetus. These adaptations are aided by vasodilators like nitric oxide, prostaglandins, progesterone, estrogen, and relaxin, which help with blood vessel widening and remodeling [10]. Vasodilation is accompanied by a decrease in systemic vascular resistance (SVR), which drives the activation of the renin–angiotensin–aldosterone system (RAAS). Subsequently, this leads to sodium and water retention and increased blood volume [11].

Key adaptations include a reduction in SVR and an increase in cardiac output, heart rate (HR), and stroke volume, as well as a rise in plasma volume and red blood cell volume. The vascular system undergoes remodeling, characterized by increased compliance and reduced arterial stiffness, to support enhanced uteroplacental blood flow [10]. Uteroplacental shunting and vasodilation, which occur secondary to a resistance to vasopressors, further contribute to the decrease in SVR. Cardiac output increases by up to 50% by 20 to 24 weeks of pregnancy, primarily driven by increased preload due to a rise in blood volume, elevated HR, and stroke volume and a reduction in SVR, which lowers afterload [10]. The decline in SVR additionally promotes fluid and sodium retention by triggering activation of the RAAS. The decrease in SVR increases HR through baroreceptor-mediated sympathetic system activation, resulting in a HR increase of up to 30 beats per minute (bpm), peaking at an average of 91 bpm by 34 weeks [12].

From the first trimester, systolic, diastolic, and mean arterial blood pressures decrease, reaching their lowest point at around 18–20 weeks, and then increase during the third trimester. In late pregnancy, blood pressure may exceed pregestational levels. By 34 weeks of gestation, plasma volume increases by 40%–50% compared to normal levels. Hematopoiesis and red blood cell volume increase by 25% above baseline values by 34 weeks. The disparate increase in plasma volume results in the dilutional anemia of pregnancy, contributing to decreased blood viscosity and increased cardiac output [10].

The increased synthesis of coagulation factors and fibrinogen along with the decrease in antithrombin III and free protein S levels and lower rate of fibrinolysis results in a hypercoagulable state, which is critical in preventing excessive postpartum hemorrhage [13]. Other changes include an increase in renal blood flow by 50%–80% to facilitate the removal of metabolic waste from both the mother and the fetus. Enhanced renal blood flow is critical for the increased excretion of maternal and fetal waste products, maintaining fluid and electrolyte balance [12]. Tidal volume and minute ventilation increase due to progesterone’s stimulatory effect on the respiratory center, leading to slight respiratory alkalosis, improving maternal oxygenation and carbon dioxide removal [14]. A summary of the cardiovascular changes seen during a normal pregnancy is presented in Table 1.

## 4. Effect of cardiovascular disorders on maternal and fetal health

About 17% of pregnancy-associated maternal deaths are attributed to cardiac conditions [15]. The prognosis for both mother and fetus depends largely on the specific type and severity of the heart disease. The perinatal period, from the early third trimester to the postpartum period, carries the highest risk of cardiac events [2,16].

Fetal complications such as intrauterine growth restriction, preterm birth, and low birth weight are common. Additionally, the risk of miscarriage and fetal hypoperfusion with consequent hypoxemia especially in cyanotic heart disease is increased. In heart diseases necessitating anticoagulation, teratogenic and hemorrhagic risks exist for the fetus [17]. Finally, parental congenital heart disease can increase the risk of inherited heart disease in the fetus with differing rates of penetrance depending on the specific defect [18].

## 5. Differentiating normal pregnancy changes from heart disease

The cardiovascular changes that occur during pregnancy can mask underlying heart diseases or mimic symptoms of cardiac dysfunction. This overlap can lead to delayed diagnosis of serious conditions such as peripartum cardiomyopathy or aortic dissection [17]. Physical examination combined with diagnostic tools, such as electrocardiography (ECG), transthoracic echocardiography (TTE), and magnetic resonance imaging (MRI) (ideally performed without gadolinium), along with serial measurements of biomarkers such as natriuretic peptides, can be used for differentiating normal physiological changes from pathological conditions. Increased values of N-terminal pro-B-type natriuretic peptides may indicate cardiac stress or heart failure (HF) [19]. Cardiopulmonary exercise testing may also be employed. Other tests involving ionizing radiation must be avoided unless absolutely necessary. Lead shielding should be used to decrease fetal radiation exposure.

On physical examination, there are a number of findings seen in normal pregnancies that may also be seen with heart disease. Dyspnea, tachycardia, varicose veins, pedal edema, and increased jugular venous pressure may be normal physiologic findings in pregnancy. However, findings such as worsening dyspnea, orthopnea, paroxysmal nocturnal dyspnea, nightly cough, hemoptysis, chest pain, cyanosis, unrelenting dilation of neck veins, cardiomegaly, presence of diastolic murmurs, systolic murmurs > 2/6, and consistently split second heart sound may be suggestive of a cardiac disturbance [17].

Due to hyperdynamic circulation during pregnancy, a systolic flow murmur can be heard in up to 90% of pregnant patients and S3 can be auscultated in over 80% of patients [20]. Additionally, auscultation findings during healthy pregnancies include increased intensity and splitting of S1, systolic ejection murmur, mammary souffle, especially on the left side, and venous hum suprasternally. The apical impulse may be displaced 2–3 cm lateral to its prepregnancy position. Rarely, an early diastolic murmur can be heard [17].

Some suspicious features of ECG in pregnant women can be accepted as normal if asymptomatic [21]. Left deviation of the QRS axis, increased frequency of prominent Q waves, and abnormal T waves including T wave inversion and flat T-waves were significantly increased in normal pregnancies.

Among the echocardiographic changes that are considered normal in pregnancy are increased left ventricular end diastolic dimension, left ventricular mass, cardiac output, right ventricular diastolic area, increased valvular regurgitation, especially tricuspid and pulmonic, increased right and left atrial size, increased valvular annulus dimension, and increased aortic and pulmonary velocity time integral [22].

## 6. Cardiac risk stratification and counseling

Various tools are employed to predict cardiovascular complications during pregnancy. The mWHO [1,23–25], CARPREG II (Cardiac Disease in Pregnancy II) [26], and ZAHARA II (Pregnancy in Women with Congenital Heart Disease II, translated from Dutch) [27] are among the most commonly used risk classification systems.

The mWHO classification (Table 2) categorizes women into four risk classes according to their cardiovascular condition [1,23,28].

CARPREG II provides a comprehensive risk assessment by generating a risk score, with one point assigned for the presence of each variable, such as prior arrhythmia or cardiac events, baseline New York Heart Association (NYHA) [29] class > II, mild or moderately obstructed left heart, and ventricular dysfunction with ejection fraction (EF) < 40%. A CARPREG II risk score of 0 carries minimal risk (less than 5% of unfavorable events), a score of 1 corresponds to a 27% risk, and a score higher than 1 indicates a 75% risk, which may necessitate pregnancy termination [26,28].

The ZAHARA II risk scoring system considers several factors in addition to those included in the CARPREG risk scoring. Besides prior arrhythmic events, preexisting NYHA class > II, and left heart obstruction, it also includes moderate to severe atrioventricular valve regurgitation, pulmonary valve regurgitation, history of heart medication, and the presence of a mechanical prosthetic valve [27]. Meanwhile, ZAHARA II is particularly effective in CHD, incorporating Doppler and echocardiographic measures to assess uteroplacental flow, although it may overestimate risk in lower-risk pregnancies [30].

A prospective study by the ZAHARA researchers demonstrated that the mWHO risk classification was superior to the CARPREG II and ZAHARA II risk scoring systems [30]. The mWHO risk stratification is recommended for risk assessment by both American and European professional cardiac societies [1,31,32]. Although the mWHO risk stratification system is considered the most accurate, it may be better suited for high-income rather than low-income countries [1].

Among the available risk stratification models for cardiovascular disease in pregnancy, the mWHO classification consistently demonstrates superior predictive accuracy, particularly for CHD, with an AUC ranging from 0.75 to 0.83 across various studies [33]. The ZAHARA II model performs well specifically in CHD cases, but may overestimate risk in lower-risk pregnancies [34]. CARPREG II, while useful for general cardiac conditions, is shown to have lower accuracy in congenital cases, with an AUC of about 0.66–0.73 [35].

## 7. Congenital heart disease

CHDs are the most prevalent heart disorders among pregnant women in high-income countries. Advances in medical and surgical treatments have enabled many women with complex CHDs to reach childbearing age.

A recent study from the ESC EORP ROPAC Registry, which included data from 53 countries, found that out of 5739 pregnancies with various forms of heart diseases, 3295 (57.4%) women had CHD, indicating a significant prevalence of CHD among pregnant women with heart diseases [36]. Furthermore, a metaanalysis that included data from six studies with a total of 3426 pregnancies found that in high-income countries CHD is the most common cardiovascular disorder affecting pregnancy, accounting for around 80% of cases [37]. Additionally, the number of women with CHD becoming pregnant is increasing due to advancements in surgical care, further increasing the prevalence of CHD in this population [38].

CHDs can vary from simple defects like atrial septal defects (ASDs) to complex anomalies such as tetralogy of Fallot (TOF) and Eisenmenger syndrome (ES). Women with uncorrected CHD face higher risks of maternal mortality and HF compared to those with corrected CHD. According to the ESC EORP ROPAC Registry, women with uncorrected CHD had maternal mortality rates of 0.7% and HF rates of 8.7%, with ES posing particularly high risks (65.5% for cardiac events and 10.3% for maternal mortality) [36].

### 7.1. Acyanotic congenital heart disease

The majority of CHDs in pregnant women are acyanotic defects, including those with left-to-right intracardiac shunting, such as ASD, VSD, and patent ductus arteriosus (PDA). ASDs and VSDs are the most prevalent congenital heart defects, with left-to-right shunting lesions comprising approximately 44.7% of uncorrected CHD cases in pregnant women [36]. These shunts cause blood to flow from the left side of the heart to the right, leading to increased pulmonary blood flow and potential complications like PAH, volume overload, HF, and arrhythmias. The risk of adverse outcomes is particularly high in women with large, uncorrected shunts or those with associated PAH.

### 7.2. Cyanotic congenital heart disease

Right-to-left shunts, present in conditions like TOF, total anomalous pulmonary venous return (TAPVR), transposition of the great arteries, truncus arteriosus, tricuspid valve abnormalities seen in Ebstein’s anomaly (EA), and ES, allow deoxygenated blood to bypass the lungs and enter systemic circulation. This results in hypoxemia and cyanosis. Symptoms may include severe shortness of breath, fatigue, and cyanosis, which can worsen during pregnancy due to increased blood volume and cardiac output.

Maternal right-to-left shunting carries high risk for both maternal and fetal complications, including HF, arrhythmias, preterm birth, and intrauterine growth restriction. Chronic maternal cyanosis increases the risk of both hemorrhage and thrombosis due to hemostatic abnormalities [39]. The prognosis largely depends on the kind and degree of severity of the defect, as well as the presence of any corrective surgery performed prior to pregnancy. Women with surgically corrected TOF generally have a better prognosis compared to those with untreated conditions [39].

Effective management strategies include preconception counseling, regular echocardiographic assessments, and use of phosphodiesterase inhibitors and prostacyclin PGI2 analogues such as iloprost [40].

## 8. Acquired heart disease

Acquired heart diseases in pregnancy include conditions that develop over a woman’s lifetime, such as hypertensive disorders, coronary artery disease, and cardiomyopathies. Cardiovascular disease, especially in cases involving hypertensive disorders and acute coronary syndromes, is the leading cause of maternal mortality [2]. Furthermore, women with a history of RHD may encounter significant challenges during pregnancy. A study conducted at a tertiary care hospital in India reported that 19.05% of pregnant women with cardiac conditions had isolated mitral stenosis associated with RHD, which was associated with high rates of maternal complications, including HF and postpartum cardiac events [41].

### 8.1. Hypertensive disorders

Hypertensive disorders of pregnancy (HDP) include chronic hypertension, gestational hypertension, preeclampsia, and eclampsia, affecting approximately 5%–10% of pregnancies globally [4,5]. A metaanalysis reported a pooled prevalence of HDP at 6.82%, with preeclampsia accounting for 4.74% of cases [42]. HDP are defined by elevated blood pressure and, in the case of preeclampsia, the presence of proteinuria and/or end-organ dysfunction. The pathophysiology involves complex interactions among genetic, environmental, and immunological factors, driving endothelial dysfunction and impaired placental perfusion [43].

In patients with preexisting heart disease, the prevalence of preeclampsia (14.3%) is significantly higher than in those without heart disease (2%–3%) [44]. It is thought that preexisting or unmasked heart disease in pregnancy can trigger preeclampsia through disturbances in vascular remodeling and endothelial dysfunction. Hypertensive disorders pose immediate risks in pregnancy and long-term risks for cardiovascular health, including higher chances of hypertension, heart disease, and stroke [45].

Pregnant women with HDP may experience symptoms such as headache, visual disturbances, epigastric pain, and edema. Severe cases can lead to complications like placental abruption, preterm delivery, and intrauterine growth restriction. Management of HDP involves close observation of maternal and fetal well-being, lifestyle modifications, and pharmacologic interventions, including antihypertensive medications and low-dose aspirin for high-risk women [46].

### 8.2. Coronary artery disease

Coronary artery disease (CAD) during pregnancy, though uncommon, poses significant risks to both the mother and the fetus. Physiological changes during pregnancy, such as increased blood volume and cardiac output, can worsen existing coronary conditions, potentially leading to acute myocardial infarction (AMI). The incidence of pregnancy-associated AMI is up to four times higher than in age-matched nonpregnant women [47]. The risk of AMI increases with maternal age, varies with ethnicity, and is particularly higher in the third trimester. AMI most frequently affects the anterior myocardium.

Managing CAD in pregnant women requires balancing both maternal and fetal well-being. Initial medical management includes antiplatelet agents, aspirin, and beta-blockers. Statins should generally be avoided. Invasive procedures such as percutaneous coronary intervention (PCI) are considered for acute coronary syndromes. The timing of interventions is critical, with the second trimester being the safest period for invasive procedures to minimize radiation exposure and teratogenic risks. Post-PCI management typically includes dual antiplatelet therapy with a preference for bare-metal stents to reduce the duration of required anticoagulation therapy [48]. Vaginal delivery is generally preferred and delivery should be avoided within 2 weeks of AMI [47].

### 8.3. Spontaneous coronary artery dissection

Spontaneous coronary artery dissection (SCAD) is the major cause of myocardial infarction in pregnant women. It often occurs in the peripartum period and is characterized by the separation of the coronary artery walls, leading to reduced blood flow and myocardial ischemia. SCAD is frequently related to fibromuscular dysplasia. Diagnosis is challenging and requires high clinical suspicion, often confirmed through coronary angiography [49].

Management of SCAD in pregnancy focuses on conservative treatment to avoid the risks associated with invasive procedures. Beta-blockers are commonly used to manage symptoms and prevent further dissection. When conservative management is inadequate, revascularization through PCI or coronary artery bypass grafting may be necessary [50].

## 9. Specific heart diseases based on the mWHO risk categories

### 9.1. mWHO class I cardiac disease

The rate of maternal cardiovascular events in this class is minimal, estimated between 2% and 5% with no significant increase in maternal mortality [1,23] (Table 2).

#### 9.1.1. Atrial septal defect

Ostium secundum ASD constitutes the major type of ASD, accounting for about 75% of all ASDs diagnosed in adulthood. It is also the most common CHD in pregnancy. This defect often remains asymptomatic until adulthood or pregnancy. Corrected ASDs carry minimal risk during pregnancy. However, in unrepaired ASDs accompanied by arrhythmias, right heart dilation, and pulmonary hypertension, the risk of adverse events in pregnancy increases. For example, a recent study showed that 44% of adult patients with ostium secundum ASD had mild to moderate pulmonary hypertension, which significantly impacts their cardiovascular health during pregnancy [51]. Since pregnancy poses an augmented risk of deep venous thrombosis (DVT), vigilance and preventive measures (ambulation and antithrombotic stockings) against paradoxical embolization into the systemic circulation and then to vital organs is necessary in unrepaired cases. Additionally, prevention of excessive hemorrhage is necessary as it may increase left-to-right shunting [52].

Management strategies for pregnant women with ASD II typically involve careful monitoring of the defect size and shunt severity, as large defects (>10 mm) are more likely to lead to complications such as HF and arrhythmias [53]. In some cases, percutaneous or surgical closure of the ASD before pregnancy may be recommended to reduce risks and improve outcomes. Anticoagulants may be used in high-risk patients.

#### 9.1.2. Ventricular septal defect

VSDs are also among the most prevalent congenital cardiac anomalies encountered during pregnancy. VSDs can vary significantly in size and impact. Small muscular VSDs often remain asymptomatic and may not significantly impact pregnancy outcomes; however, larger defects, particularly those complicated with PAH or ES, can lead to severe complications. For instance, studies highlighted that untreated large VSDs with ES present a high risk for maternal and fetal mortality [54].

In addition, muscular VSDs often close spontaneously either prenatally or in early childhood, reducing the need for intervention and highlighting the variability in clinical outcomes based on defect size and location [55]. There is a heart disease risk of up to 7% in offspring born to mothers with VSD [44].

#### 9.1.3. Patent ductus arteriosus

PDA is a congenital heart defect in which the ductus arteriosus, a blood vessel that bypasses the lungs in fetal circulation, remains open after birth. The severity of PDA varies, with some cases being small and asymptomatic, while others are large and hemodynamically significant. Large PDAs are particularly concerning as they can lead to HF, PAH, and ES.

Pregnant women with PDA may experience symptoms such as dyspnea, palpitations, and listlessness. In severe cases, symptoms of HF may also occur.

The prognosis for pregnant women with PDA depends significantly on the size of the defect and the presence of any associated pulmonary hypertension or HF. Small, asymptomatic PDAs typically do not pose a major risk during pregnancy and can often be managed conservatively. In contrast, large PDAs can lead to severe complications, necessitating a more aggressive management approach. Management of PDA during pregnancy typically involves close monitoring and, in some cases, medical or surgical intervention. Pharmacological closure using agents such as indomethacin or ibuprofen has been shown to be effective in some cases, although the safety and efficacy of these treatments during pregnancy require careful consideration [56]. Follow-up care with regular echocardiographic assessments is necessary to monitor the size of the ductus arteriosus and evaluate cardiac function [57].

#### 9.1.4. Anomalous pulmonary venous return

Although corrected anomalous pulmonary venous return (APVR) carries minimal risk of adverse events during pregnancy, uncorrected APVR may lead to pulmonary arterial hypertension and result in serious maternal and fetal risk [58].

#### 9.1.5. Pulmonary valve stenosis

Isolated mild pulmonary stenosis that is not associated with other cardiac defects and repaired pulmonary stenosis do not increase the risk of adverse events in pregnancy. An increased risk of preeclampsia (14.3%), prematurity (14.5%), and perinatal mortality (4.5%) was observed among pregnant women with repaired and unrepaired pulmonic valvular stenosis. CHD was seen in 2.8% of offspring [44].

Severe pulmonic stenosis may lead to right ventricular failure, which is associated with worse prognosis in pregnancy. Percutaneous correction of pulmonic stenosis during pregnancy has been associated with improved outcome [59].

#### 9.1.6. Mitral valve prolapse

Mitral valve prolapse (MVP) has a good prognosis during pregnancy [60]. MVP is not always associated with regurgitation and, in the absence of other cardiac disorders, patients may expect a normal pregnancy. However, MVP may be associated with progressive mitral regurgitation, arrhythmias, infective endocarditis, and transient ischemic attacks. Surgical interventions may be necessary and beta-blockers may be used but should be used carefully during pregnancy.

### 9.2. mWHO class II cardiac disease

This class includes cardiac disease carrying a slight increased risk of maternal mortality or moderately increased risk of morbidity with cardiac event risk ranging between 6% and 10% [1,23] (Table 2). Other specific conditions in this class were described in the previous section.

#### 9.2.1. Tetralogy of Fallot

TOF is the most prevalent form of cyanotic CHD, representing up to 10% of congenital heart conditions [39,61], and is commonly encountered in pregnant women. This condition is characterized by four structural defects: right ventricular hypertrophy, VSD, pulmonary stenosis, and overriding aorta. The risk of congenital anomalies in offspring is significantly increased, particularly with 22q11.2 deletions.

TOF often requires prior surgical correction, but residual defects and associated pulmonary regurgitation or right ventricular dysfunction can pose risks even in corrected cases. A review highlighted that women with TOF and transposition of great arteries have maternal mortality rates ranging from 3% to 10% in severe cases [62]. When TOF has been surgically corrected, the outcome of pregnancy is generally good, although patients may experience increased morbidity due to pulmonary valve insufficiency, leading to right ventricular dysfunction. Complications can occur, particularly in patients with severe pulmonary regurgitation [63]. The primary forms of arrhythmias include premature beats and supraventricular arrhythmias, which can be managed with beta-blockers. In rare instances, direct current cardioversion can be used without significant risk to the mother or fetus [39]. For atrial fibrillation, low molecular weight heparin (LMWH) should be initiated until birth. In rare cases of HF in repaired defects, diuretics and hydralazine can be administered and delivery can be expedited.

The majority of pregnancies in women with repaired TOF are successful; however, careful monitoring is necessary, particularly in the late postpartum period [64]. Vaginal delivery is preferred due to its minimal hemodynamic impact and passive descent of the fetal head is recommended. Regional anesthesia is also advised [39].

In contrast, pregnancy is not recommended for patients with unrepaired TOF. During pregnancy, the decrease in SVR exacerbates right-to-left shunting, leading to severe hypoxemia, especially during exertion and can be life-threatening. These patients are also at an increased risk of HF as the pregnancy progresses.

#### 9.2.2. Supraventricular arrhythmias

Pregnancy produces a proarrhythmogenic state, and arrhythmias are the most common cardiac disturbances during this period, affecting women both with and without structural heart disease. The physiological changes during pregnancy, such as increased HR and cardiac output, predispose women to arrhythmias, including supraventricular tachycardia (SVT), atrial fibrillation (AF), and, less commonly, ventricular tachycardia (VT). Factors contributing to this predisposition include increased HR, myocardial stretch due to increased plasma volume, and increased catecholamine sensitivity. Stretch-mediated ion channel activation triggers membrane depolarization, further contributing to arrhythmias [2]. Risk factors include pregestational heart disease.

Medication for arrhythmias during pregnancy includes beta-blockers, calcium channel blockers, and, in refractory cases, antiarrhythmic drugs like amiodarone. Catheter ablation may be used for drug-refractory arrhythmias that affect hemodynamic stability, with fluoroscopy avoided to minimize radiation exposure [65]. Delivery can potentially ameliorate arrhythmias.

Supraventricular arrhythmias, particularly AF or atrial flutter, are the most commonly encountered arrhythmias in pregnant women. Underlying heart disease should be investigated in cases of AF, as it is rare in women of reproductive age without structural heart disease. Common risk factors include preexisting AF and mitral valvular disease, particularly mitral stenosis. Hyperthyroidism and electrolyte imbalances can also trigger AF. Symptoms include palpitations, dizziness, and syncope. AF increases the risk of thromboembolic events, including stroke, and can exacerbate HF symptoms [65].

Management of AF typically includes rate control with beta-blockers or digoxin and anticoagulation to reduce stroke risk. Rhythm control with antiarrhythmic drugs may be necessary in some cases [66]. In hemodynamically stable patients, electric or pharmacological cardioversion can be attempted, though the potential effects of antiarrhythmic drugs on the fetus must be considered. These drugs cross the placenta and should be avoided, particularly in the first trimester. Beta-blockers, calcium channel blockers, and cardiac glycosides are the initial treatments of choice. For hemodynamically unstable patients, direct current electrical cardioversion should be performed.

As in nonpregnant adults, pregnant women with AF lasting longer than 48 h or of unknown duration should receive anticoagulation for 4 weeks before and after cardioversion [67]. Prophylactic LMWH is preferred but should be withheld before delivery or anesthesia. Warfarin is generally avoided in the first trimester and later third trimester due to increased bleeding risk. The route of delivery should be based on obstetric indications.

### 9.3. mWHO class II to III cardiac disease

Depending on individual risk factors, the conditions in this class can carry class II or III risk with maternal cardiac event risk ranging between 10% and 19% [1,23] (Table 2). Other specific conditions in this class were described in the previous sections.

#### 9.3.1. Left ventricular dysfunction with ejection fraction over 45%

Research indicates that pregnant women with mild left ventricular dysfunction (EF > 45%) generally tolerate pregnancy well, although there is an increased risk of HF and arrhythmias [30]. Despite these risks, maintaining a relatively high EF fraction typically suggests a better prognosis compared to those with more severe impairment [68]. Echocardiographic monitoring during pregnancy is essential to evaluate any decline in cardiac function, especially during the third trimester and the postpartum period when hemodynamic changes are most pronounced. The route of delivery should be based on obstetric indications.

#### 9.3.2. Hypertrophic cardiomyopathy

Hypertrophic cardiomyopathy (HCM) is the leading genetic heart disease. Preexisting HCM increases the risk of sudden cardiac death, arrhythmias, and HF. Decreases in SVR can negatively impact the left ventricular outflow tract (LVOT) gradient. Changes in contractility, HR, and vascular volume can significantly affect patients with HCM, especially those with left ventricular obstruction. HF may be triggered by the relatively short diastolic filling time due to increased contractility and HR.

Although maternal and fetal outcomes in pregnancies with HCM are generally favorable, there is a notable rate of maternal HF (15%) and arrhythmias (11.7%). Maternal mortality remains low (0%–2%), but prematurity occurs in 26% of pregnancies with HCM [69].

Symptomatic pregnancies can be managed with beta-blockers or calcium channel blockers. During labor and delivery, diastolic blood pressure should be maintained above 70 mmHg. Vaginal delivery is preferred. It is important to avoid hypovolemia, decreases in SVR, and medications that increase ventricular contractility, as these can exacerbate LVOT obstruction.

#### 9.3.3. Native or bioprosthetic valvular heart disease

Native valvular heart disease remains an important cause of morbidity and mortality, arising from congenital or acquired disease. Rheumatic valvular heart disease is the leading cause of mortality among children and young adults, particularly women, in low- and middle-income regions. It is also the most common cause of heart disease during pregnancy in these areas [70]. For patients with valvular heart disease, vaginal delivery with regional anesthesia is generally preferred, with circumvention of the Valsalva maneuver. However, caesarean delivery may be recommended in advanced aortic stenosis.

#### 9.3.4. Rheumatic heart disease

RHD remains a significant concern during pregnancy, especially in low-income countries where the prevalence of rheumatic fever is higher. This condition results from chronic damage to the heart valves due to repeated episodes of acute rheumatic fever, an inflammatory disease caused by group A streptococcal infections. Common manifestations include mitral stenosis, mitral regurgitation, and aortic valve involvement, which can lead to HF, AF, and thromboembolic events [70].

#### 9.3.5. Mitral stenosis

Mitral stenosis (MS), a reduction in the mitral valve opening, is commonly caused by RHD. This condition poses significant risks during pregnancy due to the increased hemodynamic burden. Pregnant women with MS often experience symptoms such as dyspnea, fatigue, and palpitations, which can be exacerbated with advancing gestation. The prognosis for pregnant women with MS varies according to the degree of the stenosis and the presence of comorbid conditions. Severe MS can lead to consequences such as PAH, HF, and AF. Management of MS during pregnancy typically involves careful monitoring and use of beta-blockers and diuretics to manage symptoms. In severe cases, percutaneous balloon mitral valvuloplasty may be considered to relieve MS, preferably during the second trimester to minimize fetal risks [70,71]. A study reported that 25% of patients with severe MS required percutaneous mitral valvuloplasty during pregnancy [70,71]. Regular echocardiographic assessments are necessary to monitor valve function. Postpartum, these patients should continue to be closely monitored for complications such as HF and recurrent AF.

#### 9.3.6. Mitral regurgitation

Mitral regurgitation (MR) involves the backflow of blood from the left ventricle into the left atrium due to a dysfunctional mitral valve, leading to volume overload, pulmonary congestion, and HF, especially during pregnancy. A study found that 56.6% of pregnant women with valvular heart disease had MR, with many requiring medical management to control symptoms and prevent complications [72].

Symptoms of MR may include dyspnea, fatigue, and palpitations. The prognosis for pregnant women with MR depends on the degree of regurgitation and the presence of other cardiac conditions. Management typically includes beta-blockers to reduce HR and improve symptoms and diuretics to manage pulmonary congestion. In severe cases, surgical intervention may be necessary either during pregnancy or postpartum [72]. Echocardiographic evaluations are essential.

#### 9.3.7. Aortic valve involvement

Aortic stenosis (AS) and aortic regurgitation (AR) can complicate pregnancy due to the increased cardiac workload. Pregnant women with AS may experience symptoms like dyspnea, chest pain, and syncope, whereas those with AR may present with dyspnea and fatigue. The prognosis for these patients depends on the severity of the valve disease and the presence of left ventricular dysfunction. Management strategies include close monitoring, beta-blockers, and diuretics. In severe cases, valve replacement surgery may be considered, although it carries significant risks during pregnancy [73]. Follow-up care involves regular echocardiographic monitoring of valve left ventricular performance [73].

#### 9.3.8. Thromboembolic events

Thromboembolic events pose a significant risk in pregnant women with heart disease and particularly in those with RHD, especially those with AF or severe MS. These events can include DVT, pulmonary embolism PE, and stroke. Management typically involves anticoagulation therapy to prevent thrombus formation and manage existing thrombi [74]. The predictive scoring system developed by Baghel et al. can help identify high-risk patients and guide management during pregnancy [74].

#### 9.3.9. Ebstein’s anomaly

EA is a rare CHD characterized by structural abnormality of the tricuspid valve and right ventricle, leading to different degrees of tricuspid valve regurgitation and right atrial enlargement. The prognosis for pregnant women with EA depends on the severity of tricuspid valve dysfunction and associated complications such as cyanosis and arrhythmias. Symptoms can be mild or may be associated with hypoxemia and HF. According to a study, 9.9% of pregnant women with EA experienced major adverse cardiac events, including HF and arrhythmias [75].

Complications during pregnancy for women with EA can be severe, including preterm delivery, fetal growth restriction, and neonatal CHD. Regular echocardiographic monitoring is essential to evaluate tricuspid valve function and right ventricular size and function. Pharmacological management may involve beta-blockers to control HR and prevent arrhythmias. In some cases, surgical interventions such as tricuspid valve repair or replacement may be necessary before or during pregnancy [76].

#### 9.3.10. Prosthetic heart valves

Managing pregnancy in women with prosthetic heart valves presents significant challenges due to the thrombogenic state of pregnancy and the need for effective anticoagulation. Prosthetic heart valves can be classified as mechanical and bioprosthetic valves, each with distinct implications in pregnant women. Anticoagulation throughout life is necessary in the case of mechanical valves and it poses risks of bleeding and teratogenicity. Conversely, bioprosthetic valves, while eliminating the need for lifelong anticoagulation, have a limited lifespan and can deteriorate faster during pregnancy due to hemodynamic stress [77]. The prognosis for pregnant women with prosthetic heart valves largely depends on the type of valve, adequacy of anticoagulation, and the presence of any complications such as valve thrombosis or HF [78].

#### 9.3.11. Aortic coarctation

Coarctation of the aorta (CoA) is a congenital heart defect that involves reduction in the aortic lumen. Prognosis depends mainly on whether it is corrected and the degree of CoA and associated hypertension. Patients with repaired CoA may anticipate normal pregnancies but with relatively increased risk of miscarriage and preeclampsia [79,80]. On the other hand, unrepaired, residual, or recoarctation during pregnancy can be associated with severe complications. Symptoms of CoA in pregnant women may include hypertension, headaches, and leg fatigue due to decreased blood flow to the lower extremities. Severe cases can present with HF, aortic dissection, or rupture [79].

A study reported that 20% of women with CoA developed preeclampsia and 7% experienced pregnancy-induced hypertension [80]. Furthermore, adverse cardiovascular incidents such as HF and arrhythmias can occur, with a reported incidence of 4.3% in a prospective registry study of women with repaired and unrepaired CoA [2].

Management of CoA typically includes preconception counseling, rigorous blood pressure control, and regular cardiovascular monitoring using echocardiography and MRI to assess the aorta. In some cases, percutaneous interventions such as balloon angioplasty or stenting may be necessary to alleviate the aortic narrowing and improve hemodynamics [81]. Postpartum follow-up for continued surveillance for recoarctation, aneurysm formation, and other long-term complications is critical. For both repaired and unrepaired CoA, vaginal delivery with regional anesthesia is preferred.

#### 9.3.12. Marfan syndrome

Marfan syndrome (MFS) is a connective tissue disease that poses significant risks during pregnancy due to its association with aortic dilatation and the potential for life-threatening aortic dissection. Common symptoms include fatigue, palpitations, and chest pain, which may indicate underlying aortic issues. Complications in pregnant women with MFS are often severe, including aortic dissection, HF, and preterm delivery. A comprehensive review demonstrated that women with undiagnosed MFS experienced higher rates of complications associated with pregnancy, such as preeclampsia, fetal deaths, and aortic disease [82]. A study found that pregnancy-related aortic dissections occurred primarily in women who did not have previous knowledge of their condition, underscoring the importance of early detection [83].

The prognosis for pregnant women with MFS varies depending on the degree of aortic involvement and the presence of other cardiovascular complications. The risk of aortic dissection increases significantly in the third trimester and postpartum period, with an aortic event rate between 3% and 8% during pregnancy [84].

Management strategies for MFS during pregnancy emphasize preconception counseling, rigorous cardiovascular monitoring, and the use of beta-blockers to mitigate the risk of aortic complications. Surgical interventions, such as prophylactic aortic repair, may be indicated for women with significant aortic enlargement before conception [85].

Regular imaging studies, such as echocardiograms and MRI, are essential for monitoring aortic size and assessing the risk of dissection. Postpartum follow-up is equally important to monitor for late complications.

Aortic dissection is a rare but potentially fatal event that can occur during pregnancy. The increased blood volume and cardiac output associated with pregnancy can exacerbate stress on the aortic wall, leading to dissection. In patients with MFS, an aortic root diameter above 40–50 mm and/or a rapid rise in the size of the aortic root of ≥3 mm per year is associated with a higher risk of type A aortic dissection and other cardiovascular complications [86]. Symptoms typically include sudden, severe chest or back pain, and prompt diagnosis through imaging studies like echocardiography or CT angiography is critical for timely intervention [48]. A case report highlighted the successful management of acute aortic dissection in a pregnant woman with MFS, emphasizing the need for coordinated care and timely surgical intervention [87]. Management also involves blood pressure control with beta-blockers and, in severe cases, surgical intervention.

Bicuspid aortic valve (BAV) is a relatively common CHD, affecting up to 2% of the population [88]. Symptoms such as dyspnea, chest pain, and fatigue can be exacerbated during pregnancy. The prognosis for pregnant women with BAV is largely dependent on the degree of aortic valve dysfunction and any associated aortic dilation. Severe complications, including aortic dissection and HF, can occur, especially in women with significant aortic dilation or associated congenital defects like CoA. A study on the outcomes of pregnant women with BAV found that while most experienced no severe complications, those with an aortic diameter over 45 mm were at increased risk of adverse events [89].

Regular echocardiographic monitoring is essential to assess aortic dimensions and valve function. Beta-blockers may be indicated to control blood pressure and reduce the risk of aortic complications. In cases of severe aortic stenosis or regurgitation, surgical interventions, including valve replacement, may be necessary either during pregnancy or postpartum. A retrospective study highlighted that timely surgical interventions in high-risk patients significantly improved outcomes without maternal mortality [90].

### 9.4. mWHO class III cardiac disease

Disorders in this class pose significant maternal mortality or severe morbidity with risk of cardiac events as high as 27% (Table 2) [1,23]. Other specific conditions in this class were described in the previous sections.

#### 9.4.1. Left ventricular dysfunction (ejection fraction between 30% and 45%)

Women with an EF between 30% and 45% are at an elevated risk of arrhythmias and HF during pregnancy. Regular echocardiographic monitoring is crucial to track changes in cardiac function throughout pregnancy and the postpartum period. Despite these risks, with close monitoring and a multidisciplinary approach many women with moderate left ventricular dysfunction can have successful pregnancies. The management of these patients often involves optimizing HF therapy and addressing any comorbid conditions to minimize complications [30].

#### 9.4.2. Cardiomyopathies

Cardiomyopathies encompass a diverse group of disorders that primarily affect the myocardium, leading to structural and functional impairments. Peripartum cardiomyopathy (PPCM) is the leading type of cardiomyopathy seen in pregnancy. Other types of cardiomyopathies that may affect pregnant women include hypertrophic and dilated cardiomyopathies. The causes often include genetic predispositions, previous viral infections, and, in the case of PPCM, factors related to pregnancy such as hormonal changes and inflammation [14].

Cardiomyopathies can be accompanied by HF and arrhythmias. For example, a study on women with dilated cardiomyopathy reported that 23% experienced peripartum cardiac events such as HF and ventricular arrhythmias during pregnancy [91]. Management of cardiomyopathies during pregnancy involves close monitoring with echocardiography, the use of HF medications adjusted for fetal safety, and, in severe cases, the consideration of mechanical support or transplantation.

PPCM typically occurs in the last month of pregnancy or within 5 months postpartum. It is associated with left ventricular systolic dysfunction with no other identifiable cause of HF. The prevalence of PPCM is increased in certain populations, including African-American women, those of advanced maternal age, and those with hypertension as well as multiple gestations [14].

Symptoms of PPCM often resemble those of normal pregnancy, including shortness of breath, fatigue, and edema, which can delay diagnosis and treatment. The prognosis of PPCM varies; some women fully recover cardiac function, while others may experience ongoing left ventricular dysfunction. A study found that the degree of left ventricular enlargement and systolic dysfunction at diagnosis are strong predictors of outcomes, including cardiac function recovery and mortality [92].

PPCM management includes HF treatments, adjusted to ensure fetal safety when necessary. This includes beta-blockers, diuretics, and ACEIs or ARBs in the postpartum period. Bromocriptine has shown promise in improving outcomes by inhibiting prolactin release, which may play a role in the pathogenesis of PPCM. Women with PPCM considering future pregnancies should receive preconception counseling due to the substantial risk of relapse and potential for severe complications [93].

#### 9.4.3. Systemic right ventricle

Pregnancy in women with a systemic right ventricle (sRV) following CHD corrections, such as the atrial switch performed for TGA and congenitally corrected TGA, poses significant risks but can be managed with favorable outcomes. Most women with an sRV tolerate pregnancy well, though they have an increased risk of HF and arrhythmias, which are the most common adverse events during pregnancy [94]. Predictors of adverse events include preexisting signs of HF and an sRV ejection fraction below 40% [94]. Despite these risks, studies have shown no significant deterioration in sRV function before or after pregnancy [94].

#### 9.4.4. Fontan circulation

Pregnancy in women with Fontan circulation, a palliative surgical approach for single-ventricle CHD, carries significant maternal and fetal risks. Women with Fontan circulation are at increased risk for arrhythmias and HF. Thromboembolic events are also a concern, necessitating careful management of anticoagulation therapy [95]. Hematological complications, such as thromboembolic and hemorrhagic events, are common [96]. Neonatal outcomes often include prematurity and growth restriction, with a high rate of small-for-gestational-age newborns [91].

### 9.5. mWHO class IV cardiac disease

Conditions in this group pose a maximally elevated risk of maternal mortality and/or dire morbidity. Contraindication to pregnancy exists due to the 40%–100% risk in adverse events (Table 2) [1,23]. Other specific conditions in this class were described in the previous sections.

#### 9.5.1. Systemic ventricular systolic dysfunction (ejection fraction < 30%)

HF is a major cause of morbidity and mortality in pregnancy, often due to PPCMP. However, patients with preexisting cardiomyopathies or ventricular dysfunction can experience worsening cardiac function during pregnancy. Prepregnancy risk assessment and cardiac evaluation are essential for patients wishing to conceive. High-risk factors for developing HF include preexisting HF, an EF less than 30%–40%, cardiomyopathy, pulmonary hypertension, and cardiac disease classified as NYHA class >II or mWHO class IV. Pregnancy is generally not recommended for women with these risk factors. Due to the potential for cardiac deterioration, pregnancy termination may be offered. Additionally, pregnancy-related factors such as preeclampsia, chronic kidney disease, gestational diabetes, gestational or preexisting hypertension, postpartum hemorrhage, and placental disorders can contribute to the development of HF. Preeclampsia may trigger HF in up to 30% of patients with prepregnancy structural heart disease [97]. HF is associated with an increased risk (up to eight times) of maternal mortality and a higher perinatal mortality rate (up to five times). The risks of prematurity, low birth weight, and small for gestational age are also elevated [97].

Medication adjustments for HF must be made preconceptionally for women wishing to conceive. Metoprolol and bisoprolol can be used during pregnancy, while ACE inhibitors, angiotensin receptor neprilysin inhibitors, and ARBs are teratogenic and must be avoided in all trimesters. Diuretics, digoxin, and anticoagulants, mainly in the form of vitamin K antagonists, LMWH, and unfractionated heparin, can be used during pregnancy. In advanced HF, cardiac implantable electronic devices may be necessary, although there is limited information about their use during pregnancy [98]. Vaginal delivery is preferred for most cases of HF in pregnancy. However, patients with hemodynamic instability and advanced HF may require cesarean delivery.

#### 9.5.2. Pulmonary arterial hypertension

PAH carries the biggest risk of mortality and morbidity for both the mother and the fetus. It is characterized by pulmonary artery narrowing and consequent right HF. A measurement of pulmonary arterial pressure ≥20 mmHg at rest defines PAH. The decrease in SVR during pregnancy further enhances right-to-left shunting, resulting in hypoxemia. Pregnancy must be avoided in patients with idiopathic PAH and ES.

Symptoms of ES during pregnancy include severe cyanosis, hypoxemia, and right HF, which are exacerbated by pregnancy. The prognosis for pregnant women with PAH is extremely poor, with maternal mortality rates extending between 30% and 50%, and in some cases, as high as 65% with cesarean section delivery [99,100]. ES poses extremely high risks during pregnancy, with maternal mortality rates as high as 10.3% and significant cardiac event rates of 65.5% [36]. HF, cardiac rhythm disorders, thromboembolism, and sudden cardiac death can ensue. In a study of 366 pregnancies with all types of pulmonary hypertension, maternal morbidity including HF and pulmonary hypertensive crisis was 15% and 5%, respectively. Maternal mortality reached 5.5%. Idiopathic pulmonary hypertension carried the biggest risk of maternal mortality (4/12). The incidence of preterm delivery and neonatal low birth weight was 74.6% and 78%, respectively. Fetal and neonatal mortality was 0.5% and 3.3%. The highest risk of mortality was seen between 28 weeks gestation and 1 week postpartum [16].

A case series reported that 27% of women with ES experienced maternal mortality, highlighting the critical risks involved [101]. The same study also noted complications such as preeclampsia (50%), abruption (22%), and fetal growth retardation (62.5%). Increased risk of postpartum hemorrhage may be seen with ES.

During labor, the Valsalva maneuver can raise SVR. Consequently, a decreased cardiac output can result in a significantly decreased cranial perfusion, which can be life threatening. A case report from a multidisciplinary team demonstrated the importance of this approach; a woman with ES was managed successfully through elective cesarean section at 36 weeks of gestation, resulting in a positive maternal and neonatal outcome [102]. Cesarean delivery is indicated in the majority of cases for cardiac reasons. Management of PH often involves preconception counseling and use of advanced PAH therapies such as phosphodiesterase-5 inhibitors. Endothelin receptor antagonists are strongly discouraged due to their teratogenicity. Diuretics, digoxin, vasodilators, and vasoactive drugs may be necessary in cases of cardiac failure [16]. Heparin may be initiated at 20 weeks due to the increased risk of thromboembolism.

## 10. Management strategies

Effective emergency management protocols for cardiovascular complications during pregnancy prioritize rapid intervention while balancing maternal and fetal safety. In hypertensive crises, intravenous labetalol or hydralazine is commonly used to reduce blood pressure swiftly, minimizing the risk of maternal organ damage without significant fetal compromise [48]. For acute heart failure exacerbations, diuretics and, if needed, inotropic support are recommended to stabilize maternal hemodynamics, often necessitating ICU-level care [103]. For life-threatening arrhythmias, cardioselective beta-blockers are the popular choice, and, if no response is seen, cardioversion can be performed safely during pregnancy with minimal fetal risk [104].

## 11. Obstetric management: labor and delivery

The timing of delivery in pregnant women with heart diseases is a critical aspect of obstetric management that requires careful consideration. Delivery is generally recommended at 37–39 weeks of gestation to balance the risks of prematurity with those associated with prolonged pregnancy. However, in cases of severe maternal cardiac compromise or fetal distress, earlier delivery may be necessary. Continuous fetal monitoring is essential to assess fetal well-being, especially during labor, as fetal HR abnormalities can indicate compromised uteroplacental perfusion. Noninvasive monitoring techniques, such as cardiotocography, are routinely used, and internal fetal monitoring may be considered in high-risk cases [105]. Maternal hemodynamic monitoring is equally crucial to detect early signs of cardiovascular decompensation. Advanced hemodynamic monitoring tools, such as noninvasive photoplethysmography devices, can provide real-time data on cardiac output, stroke volume, and SVR, enabling prompt intervention if necessary [106]. Oxygen can be given, especially in cyanotic patients, but its benefits are unproven.

The mode of delivery is determined by the maternal cardiac condition and obstetric indications. Vaginal delivery is generally preferred to minimize surgical risks and hemodynamic shifts. Passive fetal descent with uterine contractions and without bearing down of the mother can pose less cardiovascular risk as these maneuvers may result in reduced venous return and a subsequent decrease in cardiac output. However, cesarean delivery may be indicated in cases of severe cardiac decompensation, fetal distress, or obstetric complications [107]. Cervical ripening is preferably achieved with mechanical dilators as they cause the least cardiovascular interference. Alternatively, prostaglandin E1 can be used. Oxytocin and artificial rupture of membranes can induce labor. However, oxytocin can lead to hypotension and cardiovascular decompensation in high-risk patients. It should be administered as a slow and dilute infusion rather than in bolus form. The dose should not exceed 5 U if a bolus is necessary [2].

Preterm labor in these patients must be approached with caution. Betamimetics, commonly used to delay labor, can cause tachycardia and increased myocardial oxygen consumption, which may exacerbate heart conditions [108]. Similarly, calcium channel blockers can lead to hypotension and negatively impact cardiac output. Thus, the choice of tocolytics should be individualized. Nifedipine is usually preferred. Nonpharmacological strategies, such as bed rest and hydration, can also be beneficial in managing preterm labor in women with heart disease [1].

## 12. Anesthesia

The choice of analgesia and anesthesia is crucial in managing labor and delivery in women with heart diseases. Neuraxial anesthesia, such as epidural or spinal anesthesia, is generally preferred as it provides effective pain relief and reduces sympathetic stress responses. Neuraxial anesthesia was shown to be effective in managing labor pain without significantly impacting maternal hemodynamics. However, vigilance regarding hypotension is necessary. In certain cases, general anesthesia may be necessary, but it carries higher risks of hemodynamic instability and should be administered with caution [109].

## 13. Pregnancy termination

In women with significant heart disease, pregnancy termination might be necessary due to the high risk of maternal and fetal compromise. The decision to end a pregnancy should involve the patient and a multidisciplinary team to ensure the best outcomes. Termination methods vary depending on the gestational age. In the first trimester, vacuum aspiration or medical abortion with medications like mifepristone and misoprostol is commonly used. For second-trimester terminations, options include dilation and evacuation or labor induction with agents such as prostaglandins, especially PGE1, which have no significant adverse cardiovascular effects. Mifepristone may also be used. It is crucial to provide effective analgesia and to monitor maternal hemodynamics closely during the procedure to prevent cardiovascular decompensation [109].

## 14. Prophylactic antibiotics

Currently, there are no recommendations for antibiotic prophylaxis during labor and delivery, even for women with high-risk heart disease. However, antibiotics administered prior to cesarean delivery to prevent endometritis also provide coverage against endocarditis. Although the risk of bacteremia from vaginal delivery is low, antibiotics should be considered 30–60 min before delivery for heart disease patients, especially those with prosthetic valves or cyanotic heart disease [1,2].

## 15. Preconception counseling section: fertility planning and contraception

For women with high cardiovascular risk, nonestrogen-based contraceptives are generally preferred to avoid potential complications. Progestin-only methods, such as progestin implants or intrauterine devices, are effective and safer options, as they do not carry the same risk of thrombosis associated with estrogen-containing contraceptives [14]. Long-acting reversible contraceptives are also recommended for their reliability and minimal cardiovascular impact, particularly beneficial for women at risk of venous thromboembolism or with hypertension [110].

## 16. Conclusion

This review has underscored the critical aspects of cardiovascular adaptations during pregnancy, highlighting how heart disease impacts both maternal and fetal health and emphasizing the importance of distinguishing normal physiological changes from pathological conditions. Effective preconception counseling, risk assessment, and careful obstetric management are essential to ensure favorable outcomes. Clinically, a multidisciplinary approach tailored to individual risk factors is vital for managing cardiac conditions during pregnancy. Future research should aim to close current knowledge gaps by developing more precise risk prediction models and leveraging emerging technologies to optimize care and outcomes in pregnant women with heart disease.

## Figures and Tables

**Table 1:** Key cardiovascular changes associated with normal pregnancy.

Change	Description	Clinical Significance	Time in Gestational Weeks
**Cardiac output**	Increases by 30%–50% to meet the increased metabolic demands of pregnancy. Peaks in the second trimester.	Enhanced cardiac output is crucial for supporting increased metabolic and circulatory demands.	20–24 weeks
**Heart rate**	Increases by 10–20 beats per minute due to increased sympathetic activity and hormonal influences.	Increased heart rate helps maintain adequate cardiac output despite reduced systemic vascular resistance.	20–24 weeks
**Stroke volume**	Increases by approximately 30% due to enhanced myocardial contractility and preload.	Increased stroke volume is essential for compensating for the higher blood volume and ensuring efficient circulation.	20–24 weeks
**Blood volume**	Increases by 40%–50% to provide adequate perfusion to the placenta and growing fetus.	Expanded blood volume helps meet the oxygen and nutrient demands of the fetus and supports uteroplacental circulation.	20–24 weeks
**Systemic vascular resistance**	Decreases due to the vasodilatory effects of progesterone and relaxin, reducing afterload.	Reduced systemic vascular resistance lowers afterload and facilitates increased cardiac output, essential for fetal development.	20–24 weeks
**Blood pressure**	Decreases in the first and second trimesters; then may return to prepregnancy levels or increase slightly in the third trimester.	Alterations in blood pressure reflect the body’s adaptation to maintain optimal perfusion pressure to vital organs and the placenta.	12–20 weeks
**Venous pressure**	Increases, particularly in the lower extremities, contributing to edema and varicose veins.	Increased venous pressure can lead to discomfort and complications such as varicose veins and edema, requiring careful management.	Third trimester
**Renal blood flow**	Increases by 50%–80% to facilitate the removal of metabolic waste from both the mother and fetus.	Enhanced renal blood flow is critical for the increased excretion of maternal and fetal waste products, maintaining fluid and electrolyte balance.	16–22 weeks
**Uterine blood flow**	Increases significantly to ensure sufficient oxygen and nutrient delivery to the fetus.	Significantly increased uterine blood flow is vital for fetal growth and development, highlighting the importance of adequate placental perfusion.	20–24 weeks
**Respiratory changes**	Tidal volume and minute ventilation increase due to progesterone’s stimulatory effect on the respiratory center, leading to slight respiratory alkalosis.	Increased tidal volume and minute ventilation improve maternal oxygenation and carbon dioxide elimination, crucial for both maternal and fetal health.	Throughout pregnancy, peaks at 24–28 weeks

**Table 2:** Modified WHO risk classification for maternal cardiovascular diseases [1].

Class	I	II	II to III	III	IV
Cardiac condition	Corrected ASD, VSD, PDA, APVR minor PS, PDA, MVP	Unrepaired ASD, VSD, repaired TOF, supraventricular arrhythmias	Minor LVD EF > 45%, HCMP, MS or AS (not severe), MFS without aortic dilatation, BAV with <45 mm aorta, repaired CoA	LVD with EF 35%–45%, history of PPCMP, mechanical valve, systemic right ventricle with minor ventricular dysfunction, Unrepaired CCHD, Moderate MS, severe AS without symptoms, aortic dilatation of 40–45 mm in MFS; 45–50 mm in BAV	PAH, systemic ventricular dysfunction with EF <30%, history of PPCM with residual LVD, Severe MS, symptomatic AS, Complicated Fontan, Systemic right ventricle with significantly depressed ventricular function, aortic dilatation (>45 mm in MFS, >50 mm in BAV, TOF > 50 mm), vascular Ehler Danlos

APVR, anomalous pulmonary venous return; PS, pulmonary stenosis; PDA, patent ductus arteriosus; MVP, mitral valve prolapse; TOF, tetralogy of Fallot; LVD, left ventricular dysfunction; EF, ejection fraction; HCMP, hypertrophic cardiomyopathy; MS, mitral stenosis; AS, aortic stenosis; MFS, Marfan syndrome; BAV, bicuspid aortic valve; CoA, coarctation of aorta; PPCMP, peripartum cardiomyopathy; CCHD, cyanotic congenital heart disease; PAH, pulmonary arterial hypertension
